# Partial correlation as a tool for mapping functional-structural correspondence in human brain connectivity

**DOI:** 10.1162/NETN.a.22

**Published:** 2025-09-19

**Authors:** Francesca Santucci, Antonio Jimenez-Marin, Andrea Gabrielli, Paolo Bonifazi, Miguel Ibáñez-Berganza, Tommaso Gili, Jesus M. Cortes

**Affiliations:** NETWORKS Unit, Istituzioni, Mercati, Tecnologie, School for Advanced Studies, Lucca, Italy; Computational Neuroimaging Lab, Biocruces-Bizkaia Health Research Institute, Barakaldo, Spain; Dipartimento di Ingegneria Civile, Informatica e delle Tecnologie Aeronautiche, Università degli Studi Roma Tre, Rome, Italy; “Enrico Fermi” Research Center – Centro Ricerche Enrico Fermi, Rome, Italy; Istituto dei Sistemi Complessi - Consiglio Nazionale delle Ricerche, Rome, Italy; Ikerbasque: The Basque Foundation for Science, Bilbao, Spain; Department of Physics and Astronomy, University of Bologna, Bologna, Italy; INdAM-GNAMPA Istituto Nazionale di Alta Matematica “Francesco Severi”, Rome, Italy; Department of Cell Biology and Histology, University of the Basque Country, Leioa, Spain

**Keywords:** Functional connectivity, Precision matrix, Partial correlation, Structural-functional coupling, fMRI BOLD resting state, Regularization

## Abstract

Brain structure-function coupling has been studied in health and disease by many different researchers in recent years. Most of the studies have estimated functional connectivity matrices as correlation coefficients between different brain areas, despite well-known disadvantages compared with partial correlation connectivity matrices. Indeed, partial correlation represents a more sensible model for structural connectivity since, under a Gaussian approximation, it accounts only for direct dependencies between brain areas. Motivated by this and following previous results by different authors, we investigate structure-function coupling using partial correlation matrices of functional magnetic resonance imaging brain activity time series under various regularization (also known as noise-cleaning) algorithms. We find that, across different algorithms and conditions, partial correlation provides a higher match with structural connectivity retrieved from density-weighted imaging data than standard correlation, and this occurs at both subject and population levels. Importantly, we also show that regularization and thresholding are crucial for this match to emerge. Finally, we assess neurogenetic associations in relation to structure-function coupling, which presents promising opportunities to further advance research in the field of network neuroscience, particularly concerning brain disorders.

## Author Summary

A precise understanding of how brain structure and function interact is fundamentally relevant to understanding disease. For the functional representation, most of the previous research has used correlation methods, which have limitations. Our study explores a different approach called partial correlation methods, which more accurately reflect the brain’s direct connections. We found that partial correlation aligns better with the brain’s structural connectivity than standard methods, both in individuals and groups. Additionally, we identified promising links between brain connectivity and genetics, offering new insights into brain disorders. Our work highlights the importance of using advanced connectivity methods to improve our understanding of the brain’s structure-function relationship, paving the way for future research in brain health and disease.

## INTRODUCTION

A fundamental problem in network neuroscience is understanding the relationship between functional connectivity (FC), accounting for similarity in the activation patterns between brain areas, and structural connectivity (SC), which maps the brain’s anatomical connections (Alonso-Montes et al., 2015; Amor et al., 2015; Bansal, Nakuci, & Muldoon, 2018; Deco, Jirsa, Mcintosh, Sporns, & Kötter, 2009; Friston, 1994; Goñi et al., 2014; Greicius, Supekar, Menon, & Dougherty, 2009; Honey, Kötter, Breakspear, & Sporns, 2007; Honey et al., 2009; Honey, Thivierge, & Sporns, 2010; Park & Friston, 2013; Sarwar, Tian, Yeo, Ramamohanarao, & Zalesky, 2021; Sporns & Betzel, 2016; Stramaglia et al., 2017; Suárez, Markello, Betzel, & Misic, 2020; van den Heuvel, Mandl, Kahn, & Hulshoff Pol, 2009; Vázquez-Rodríguez et al., 2019; Zamani Esfahlani, Faskowitz, Slack, Mišić, & Betzel, 2022). Numerous, rapidly evolving functional states emerge from the relatively static structural connectivity (Park & Friston, 2013). The underlying structure partially determines function and activity that, in turn, shapes the structure through the processes of neuromodulation and plasticity (Batista-García-Ramó & Fernández-Verdecia, 2018; Hagmann et al., 2010; Park & Friston, 2013). The investigation of the relationship between structure and function as a biomarker is generally referred to as structure-function coupling (SFC; Fotiadis et al., 2024).

It has been found that, while the intensities (*weights*) of the single connections (*links*) of the SC and FC are positively correlated at rest (Honey et al., 2009), this correlation is not always consistent and exhibits variability across individuals (Griffa, Amico, Liégeois, Van De Ville, & Preti, 2022; Gu, Jamison, Sabuncu, & Kuceyeski, 2021), ages (Gu et al., 2021; Hagmann et al., 2010), cognitive tasks (Griffa et al., 2022), brain regions (Gu et al., 2021; Preti & Van De Ville, 2019), and in brain-related disorders (Cao et al., 2020; Hua et al., 2020; Liu et al., 2022; Tay et al., 2023; Zarkali et al., 2021).

Nevertheless, the relationship between SC and FC does not follow a simple mapping at the level of the single link. For instance, two regions can be functionally connected without a direct structural connection, and SC evolves over much longer time scales than FC (Batista-García-Ramó & Fernández-Verdecia, 2018). For this reason, the SC-FC correspondence has been investigated at the module or aggregate level, exploring the full set of nested partitions within a hierarchical tree, revealing that the structural and functional connectivities share a common modular architecture (Betzel et al., 2019; Diez et al., 2015; Jimenez-Marin et al., 2024; Puxeddu, Faskowitz, Sporns, Astolfi, & Betzel, 2022; Sporns & Betzel, 2016).

FC is usually estimated as the correlation matrix between pairs of time series of activity (usually blood oxygen level-dependent [BOLD] functional magnetic resonance imaging [fMRI] signals at rest) from different brain areas and, less commonly, as the partial correlation (PC; Fransson & Marrelec, 2008; Huang et al., 2010; Peterson, Sanchez-Romero, Mill, & Cole, 2023; Rahim, Thirion, & Varoquaux, 2019).

Several advantages of PC over correlation have been shown. For instance, PC represents a more direct model for brain connectivity, since, under a linear (Gaussian) approximation, it accounts for direct dependencies between brain areas only (Liégeois, Santos, Matta, Van De Ville, & Sayed, 2020; Marrelec et al., 2006; Ryali, Chen, Supekar, & Menon, 2012; Salvador, Suckling, Schwarzbauer, & Bullmore, 2005; Varoquaux, 2019) and, accordingly, it provides a higher link-wise match between SC and FC (Liégeois et al., 2020; see also the Supporting Information for a more detailed description of the relation between structure and function in the linear approximation). Moreover, PC-based FC exhibits reduced variance across subjects (Brier, Mitra, McCarthy, Ances, & Snyder, 2015) and yields higher prediction scores for certain individual-level measures (Pervaiz, Vidaurre, Woolrich, & Smith, 2020; Rahim et al., 2019).

However, the use of the PC is limited by the low accuracy of its statistical estimation in the small-sample limit, that is, when the time series are short relative to the number of brain regions considered, which is often the case with BOLD fMRI time series (Ibáñez-Berganza, Lucibello, Santucci, Gili, & Gabrielli, 2023; Liégeois et al., 2020; Ryali et al., 2012; Varoquaux, 2019). To address this issue, various regularization methods have been proposed within network neuroscience for accurate inference of the correlation and precision matrices (Brier et al., 2015; Huang et al., 2010; Rahim et al., 2019; Ryali et al., 2012), many of which introduce ℓ_1_ or ℓ_2_ penalty terms. The ℓ_1_ penalty leads to sparse estimators, such as the Graphical Lasso (GLASSO; Friedman, Hastie, & Tibshirani, 2008), while the penalty ℓ_2_ results in linear shrinkage (LS) estimators, where matrices are not intended to be sparsified (Ledoit & Wolf, 2004; Varoquaux, 2019; Varoquaux & Craddock, 2013). The GLASSO is, in general, a good approach for structure recovery (Huang et al., 2009), but it may not yield stable covariance coefficients. Moreover, its costly optimization is not suited for large-scale datasets. On the other hand, the LS estimator is simple and fast to compute. It yields biased estimates that are more stable than the empirical covariance and is often recommended for FC (Rahim et al., 2019).

These methods have been compared in terms of nonimaging features prediction power (Pervaiz et al., 2020; Peterson et al., 2023; Rahim et al., 2019), stability across scans (Mejia et al., 2018; Peterson et al., 2023; Rahim et al., 2019), retrieval of synthetic data generative models (Peterson et al., 2023; Ryali et al., 2012; Smith et al., 2011), and measures such as test-set (or out-of-sample) likelihood (Ibáñez-Berganza et al., 2023; Varoquaux, Gramfort, Poline, & Thirion, 2010), suggesting that the choice between sparse or shrinkage estimators may depend on the intended use and interpretation of FC.

Indeed, the dependence of the SFC on the FC inference method, including PC, has already been investigated (Liégeois et al., 2020; Peterson et al., 2023), but only at the single links level, while it has been suggested that the SFC may be rooted in a common hierarchical modular organization rather than in a correspondence between the single connections (Diez et al., 2015; Jimenez-Marin et al., 2024; Puxeddu et al., 2022; Sporns & Betzel, 2016).

Here, we go beyond both approaches by exploring the correspondence between SC and FC at the hierarchical aggregated level, inferring FC from both correlation and PC matrices. Specifically, we show that (a) FC, when estimated from the regularized PC, exhibits greater similarity to SC in terms of modular structure, both at the subject and at the population level, and a smaller variance across subjects; (b) regularization is crucial to our results, which are robust with respect to the regularization methods most widely used in neuroscience; (c) sparsity is fundamental for the emergence of the FC’s hierarchical modular structure; (d) the SC-FC similarity reaches a maximum in correspondence with a specific partition of FC; and (e) the partition’s modules are characterized by neurogenetic expression present in major diseases. These findings advance our understanding of SFC in the brain, indicating that the use of regularized PC matrices may provide a more accurate and stable representation of SC, enhancing the potential for clinical applications and insights into neurogenetic disease mechanisms.

## RESULTS

### Connectivity Matrices and Cross-Modularity for Assessing the SC-FC Correspondence

**Figure 1.**
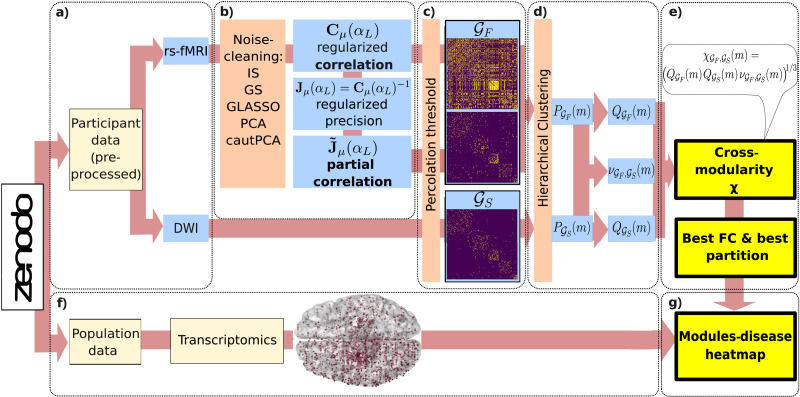
Methodological sketch and pipeline. (A) Preprocessed data of 136 healthy subjects have been obtained from the open dataset (Jimenez-Marin et al., 2024) at Jimenez-Marin et al. (2023). Data include, for each subject, a DWI matrix of *N* = 183 brain regions, and rs-fMRI BOLD time series of *N* brain regions and *T* = 652 time steps. (B) Each subject FC is estimated as (a) the regularized correlation matrix **C***_μ_*(*α_L_*) of the time series, where *μ* stands for the specific regularization method, and *α_L_* for the parameter that maximizes the validation-set likelihood, given the method; or (b) as the regularized partial correlation matrix $\tilde{J}$, the standardized version of regularized precision matrix (the correlation inverse) $J_{\mu }\left(\alpha_{L}\right)=C_{\mu }^{-1}\left(\alpha_{L}\right)$. (C) We cut both functional and structural matrices, taken in absolute value, at the percolation threshold. These thresholded matrices are the adjacency matrices of the sparse graphs $G_{F}$ and $G_{S}$. (D) We computed the hierarchical clustering of $G_{F}$ and $G_{S}$, separately. This computation returns two sets of nested partitions $P_{G_{F}}\left(m\right)$ and $P_{G_{S}}\left(m\right)$, in *m* modules, for *m* = 2, …, *N*. Subsequently, for each couple of partitions $P_{G_{F}}\left(m\right)$ and $P_{G_{S}}\left(m\right)$, we computed their individual quality ($Q_{G_{F}}\left(m\right)$ and $Q_{G_{S}}\left(m\right)$) and their agreement $\nu_{G_{F},G_{S}}\left(m\right)$. (E) These three measures allow us to compute the cross-modularity $\chi_{G_{F},G_{S}}\left(m\right)={\left(Q_{G_{F}}\left(m\right)\cdot Q_{G_{S}}\left(m\right)\cdot \nu_{G_{F},G_{S}}\left(m\right)\right)}^{1/3}$, whose maximum can be used to identify a meaningful partition of the population FC. (F) We made use of transcriptomic data extracted from Jimenez-Marin et al. (2024) and data relative to the association between genes and a set of diseases extracted from Zeighami et al. (2023) to (G) evaluate the association between each of the modules of the retrieved FC partition and these diseases.

SC graph $G_{S}$ and FC graph $G_{F}$ (or connectomes) were obtained from data previously published in Jimenez-Marin et al. (2024) and available at Jimenez-Marin et al. (2023) for a population of *P* = 136 healthy participants and *N* = 183 regions of interest (ROIs; see Figure 1 for a scheme of the methodological pipeline). In particular, we used diffusion-weighted imaging (DWI) matrices to extract a unique estimate of SC for each subject. To estimate FC, we used a resting-state (rs) fMRI signal of *T* = 652 time steps (Figure 1A). In particular, we use two differentiated strategies to estimate FC: from the correlation **C** and from the PC $\tilde{J}$ matrices, respectively. The latter is defined as ${\tilde{J}}_{ij}=\frac{J_{ij}}{\sqrt{J_{ii}J_{jj}}}$, or the standardized version of the precision matrix **J** = **C**^−1^. In the fMRI time series, cast as a *N* × *T* matrix **X**, the ratio *q* = *N*/*T* is not negligible, so regularization methods are needed to accurately estimate **C** (and even more its inverse **J**; Ibáñez-Berganza et al., 2023) beyond the sample correlation matrix $E=\frac{{XX}^{⊺}}{T}$ (the time series will be assumed from now on to be demeaned and standardized). Here, we estimated such regularized correlation matrix **C***_μ_* and regularized PC matrix ${\tilde{J}}_{\mu }$ according to different regularization algorithms *μ* (Figure 1B and the Materials and Methods section).

Next, we construct three connectivity matrices **M** for each subject, one for SC (containing the DWI data) and two for FC: *M*_*ij*_ = ∣*C*_*ij*_∣ and $M_{ij}=|{\tilde{J}}_{ij}|$, with ∣⋅∣ indicating the absolute value. Afterward, we cut the connectivity matrices at the so-called percolation threshold (Nicolini, Forcellini, Minati, & Bifone, 2020). This procedure prescribes setting to zero all matrix elements smaller than the cutoff that would break the corresponding graph in two or more connected components, so that the thresholded graphs’ density depends on their topological properties (also see the Materials and Methods section). Therefore, interpreting these thresholded matrices as adjacency matrices, SC and FC graphs were generated (Figure 1C). In the following, we will indicate the structural connectivity graph as $G_{S}$ and the FC graph as $G_{F}$, possibly distinguishing between the graphs inferred from **C** or $\tilde{J}$ as $G_{F}\left(C\right)$ and $G_{F}(\tilde{J})$, respectively. If not otherwise specified, we assume all graphs to be at the percolation threshold. Each pair of SC and FC graphs was then compared at the module level. This was done for each subject in the dataset, and at the population level as well. To this aim, we performed the hierarchical clustering of both $G_{S}$ and $G_{F}$ separately. This procedure returns two independent sets of nested partitions into *m* modules, ${\left\{P_{G_{S}}\left(m\right)\right\}}_{m=2,\ldots ,N}$ and ${\left\{P_{G_{F}}\left(m\right)\right\}}_{m=2,\ldots ,N}$. For all values of *m* = 2, …, *N*, we computed the following: (a) the quality $Q_{G_{S}}\left(m\right)$ of $P_{G_{S}}\left(m\right)$ and the quality $Q_{G_{F}}\left(m\right)$ of $P_{G_{F}}\left(m\right)$, in terms of Newman’s modularity (Newman, 2006), and (b) the agreement, $\nu_{G_{F},G_{S}}\left(m\right)$, between the pair of partitions $P_{G_{S}}\left(m\right)$ and $P_{G_{F}}\left(m\right)$, in terms of adjusted normalized mutual information (NMI; Xuan Vinh, Epps, & Bailey, 2010). Notice that, here, we did not maximize *Q* but simply calculated it at all levels in the hierarchical clustering (Figure 1D). These three quantities allowed us to compute the cross-modularity,

$$\chi_{G_{F},G_{S}}\left(m\right)={\left(Q_{G_{F}}\left(m\right)\cdot Q_{G_{S}}\left(m\right)\cdot \nu_{G_{F},G_{S}}\left(m\right)\right)}^{1/3},$$ (1)

which is a slightly different measure from the original one defined in Diez et al. (2015). The aim of cross-modularity is to quantify the reciprocal similarity of a couple of graphs in terms of their hierarchical modular structure while also taking into account the quality of their individual partitions. This is done for all number of modules, so that a complete comparison of the whole hierarchy of nested partitions of the two graphs is provided. The range of *χ*(*m*) follows straightforwardly from those of the Newman modularity and NMI: They both reach a maximum of 1 for a high-quality partition and a perfect match and are expected to vanish in case of random and unrelated partitions (Hagberg, Schult, & Swart, 2008; Pedregosa et al., 2011; Romano, Xuan Vinh, Bailey, & Verspoor, 2016; Van Mieghem, Ge, Schumm, Trajanovski, & Wang, 2010; Xuan Vinh et al., 2010). In this work, cross-modularity is always used to compare $G_{S}$ with an estimate of $G_{F}$; therefore, when referring, for the sake of shortness, to the “cross-modularity of $G_{F}$,” we will always mean the cross-modularity of between $G_{F}$ and $G_{S}$ in Equation 1.

The cross-modularity *χ*(*m*) was then used for (a) comparing the effects of estimating $G_{F}$ through **C** or through $\tilde{J}$, as well as the impact of regularization, in terms of similarity between the hierarchical nested structures of $G_{F}$ and $G_{S}$ at both the individual and population levels and (b) extracting a meaningful partition of our estimate of population-level FC graph (Figure 1E). Finally, we made use of transcriptomic data (Figure 1F; Jimenez-Marin et al., 2024) to better motivate the biological interpretation of the different estimated partitions (Figure 1G).

### PC Enhances the FC-SC Correspondence in the Human Brain

**Figure 2.**
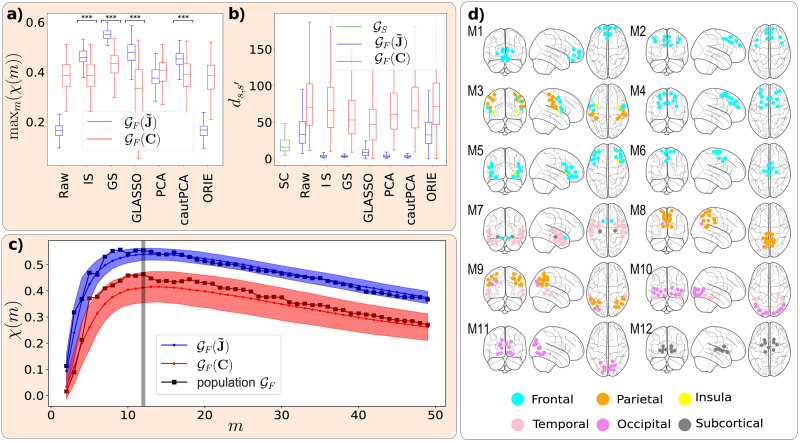
Partial correlation enhances the FC-SC correspondence in the human brain. Panels A and B show how two relevant measures such as (A) the cross-modularity and (B) the across-subject variance, measured in terms of spectral distance, change depending on whether $G_{F}$ is estimated as **C** (red) or $\tilde{J}$ (blue), and the relevance of using a regularization method (“raw” stands for no regularization). More in detail, the boxplots represent the across-subject distribution of (A) max*_m_ χ*(*m*) and (B) the spectral distances between each couple of subjects’ *d*_*s,s*′_. Most regularization strategies (denoted with three asterisks) significantly enhance the cross-modularity of $G_{F}(\tilde{J})$. Moreover, the subjects’ $G_{F}(\tilde{J})$ exhibit a significant and systematic lower spectral distance, with respect to $G_{F}\left(C\right)$, similarly to $G_{S}$, that we have reported in green for comparison. We have assessed such significance by performing a Mann–Whitney *U* rank test of the null hypothesis that the distributions underlying the pair of samples (relative to **C** and $\tilde{J}$) are the same. More in detail, in a the methods marked by the asterisks are associated with a *p* value smaller than 10^−3^, and the alternative hypothesis is that the distribution underlying the sample corresponding to **C** is stochastically *less* than the distribution underlying the sample corresponding to $\tilde{J}$. On the other hand, in b all methods are associated with a vanishing *p* value, and the alternative hypothesis is that the distribution underlying the sample corresponding to **C** is stochastically greater than the distribution underlying the sample corresponding to $\tilde{J}$. The cross-modularity curves of $G_{F}\left(C\right)$ and $G_{F}(\tilde{J})$, regularized with GS, are shown in (C), both at the subject (the solid lines are the across-subject means and the shaded areas their standard deviation) and at the population level (square solid lines). The curves of $G_{F}(\tilde{J})$ are significantly higher than those of $G_{F}\left(C\right)$ for almost all values of *m* and are characterized by a lower variance. The gray vertical line indicates the number of modules m* = 12 that maximizes the cross-modularity of most subjects. We reported in (D) the partition of the population $G_{F}(\tilde{J})$ into m* modules, drawing the ROIs belonging to each module as dots in the brain glass plots, colored according to the anatomical regions.

As represented in Figure 2, $G_{F}$ is more similar to $G_{S}$ when inferred from regularized PC than when inferred from regularized correlation, in terms of hierarchical modularity. This result is robust across all regularization strategies that are most common in neuroscience. More in detail, with reference to Figure 2A, we found that the cross-modularity *χ*(*m*) of $G_{F}(\tilde{J})$ was much lower than the one of $G_{F}\left(C\right)$ when no regularization was applied. However, when regularization was applied, $G_{F}(\tilde{J})$ reached higher values of *χ*(*m*) for most regularization methods. On the other hand, the cross-modularity of $G_{F}\left(C\right)$ did not exhibit significantly different variations after regularization (as expected). Moreover, $G_{F}(\tilde{J})$ exhibited a significantly higher *χ*(*m*) than $G_{F}\left(C\right)$, for methods such as identity shrinkage (IS), group shrinkage (GS), GLASSO, and cautious principal component analysis (cautPCA). In addition, FC inferred from PC exhibited a smaller across-subject variance. A similar trend was found measuring the spectral distance (Figure 2B) between each couple of subjects’ matrices (see the Materials and Methods section). We additionally observed that the cross-modularity of $G_{F}(\tilde{J})$ is higher when regularizing with the method cautPCA (Ibáñez-Berganza et al., 2023) than when regularizing with PCA (alternatively known as eigenvalue clipping; Bun, Bouchaud, & Potters, 2017), of which cautPCA is a variant.

### Cross-Modularity Curves Identify Representative Partitions

The complete cross-modularity curves for $G_{F}(\tilde{J})$ and $G_{F}\left(C\right)$, both for individual subjects and at the population level, are shown in Figure 2C. These curves are obtained using the GS regularization method, as it provides the highest PC cross-modularity, but we found qualitatively consistent results across regularization methods. We found that, in general, at both the individual subjects and population levels, for $G_{F}$ inferred from both regularized **C** and $\tilde{J}$, and across all regularization methods, the cross-modularity *χ*(*m*) increases rapidly with the number of modules *m* when *m* is low. It exhibits a soft maximum when *m* is between 10 and 20 (though its exact position may slightly vary depending on the specific method used for inferring FC) and then decreases slowly as *m* increases. We stress that, since the cross-modularity is the product of two quantities that are expected to vanish in unstructured (Hagberg et al., 2008; Van Mieghem et al., 2010) or unrelated graphs (Pedregosa et al., 2011; Romano et al., 2016), the fact that it presents a maximum is nontrivial, and the number of modules providing it can be used as a criterion for selecting the number of modules to partition the FC. Accordingly, Figure 2D presents the hierarchical clustering partition of $G_{F}(\tilde{J})$ into 12 modules (the median across-subject argument of max*_m_ χ*(*m*)) of the population $G_{F}(\tilde{J})$.

See the Supporting Information S1 for further details regarding the FC-SC correspondence at the population level (Supporting Information Figure S3), and the overlap between each module and the Desikan-Killiany structural areas and the Yeo functional networks (Supporting Information Figure S4C–D).

### Robustness of the Enhanced FC-SC Similarity With Respect to the Regularization Parameters

In this article, we understand *regularization* in the sense of statistical inference: the regularized estimator leads to a higher out-of-sample data likelihood at the expense of a lower training-set likelihood (Ibáñez-Berganza et al., 2023). The regularized estimator presents, in other words, a lower variance error with respect to the maximum likelihood estimator, at the expense of a higher bias error (and a lower bias + variance error). We now present an assessment of the robustness of our results with respect to the value of the regularization parameter, generically referred to as *α* for all the regularization methods. The regularization parameter is such that, for all the methods except optimally rotationally invariant estimator (ORIE), *α* = 0 represents no regularization (the maximum likelihood estimator), while *α* = 1 represents the completely biased estimator. Given a subject time series **X**, we fix the value of *α*_L_ as the one that maximizes the validation-set likelihood **X**_val_, given the method (see the Materials and Methods section).

**Figure 3.**
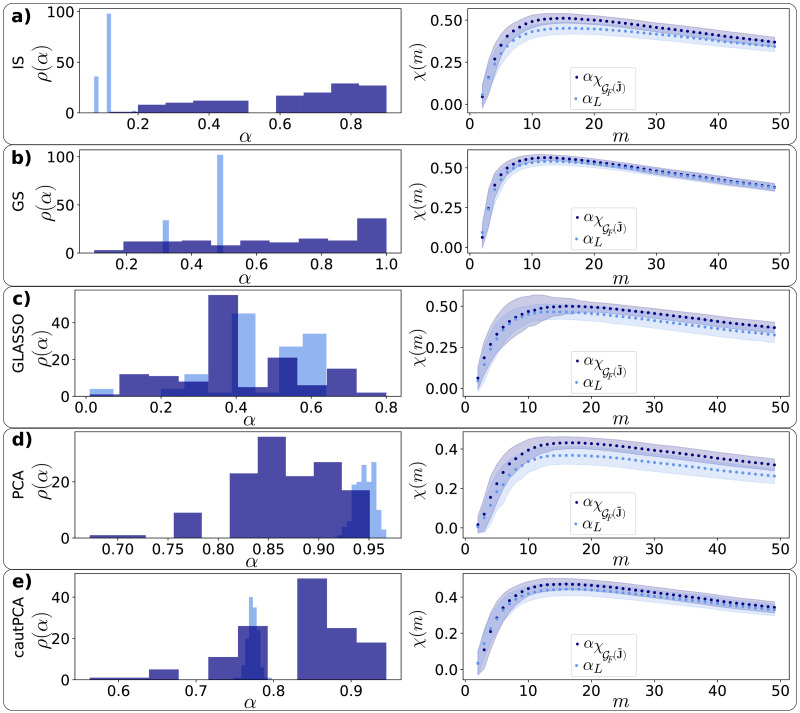
Robustness of the enhanced FC-SC similarity with respect to the regularization parameters, for different regularization methods (Rows A–E). First column: across-subjects distribution of the regularization parameter *α_L_* that maximizes the validation-set log likelihood (light blue) and the parameter $\alpha_{\chi_{G_{F}(\tilde{J})}}$ that maximizes the peak of the cross-modularity (*χ* = max*_m_ χ*(*m*)) of ${\tilde{J}}_{\mu }\left(\alpha \right)$ (dark blue), given the regularization method *μ*. Although the distributions of *α_L_* and $\alpha_{\chi_{G_{F}(\tilde{J})}}$ are different, they produce similar *χ* curves: the second column represents the cross-modularity curves of $G_{F}(\tilde{J}\left(\alpha_{L}\right))$ (light blue) and of $G_{F}(\tilde{J}\left(\alpha_{\chi }\right))$ (dark blue), where the dotted lines are the across-subject means and the shaded areas represent the plus/minus 1 *SD* distance from the mean.

We have already seen (see Figure 2A) that regularization is crucial for our estimation of PC-based FC to exhibit an enhanced similarity to SC. We now show that, actually, the enhancement of FC-SC cross-modularity induced by statistical regularization is very close to its *maximum possible value*, understood as the maximum value of the FC-SC cross-modularity over all values of *α*. Let us define *α_χ_* as the value of the regularization parameter that maximizes the (PC-based) FC-SC cross-modularity (given a regularization method). We observe (see Figure 3), that the effect of *statistical regularization* (i.e., with parameter *α*_L_) in terms of FC-SC similarity is, rather remarkably, very similar to the effect of regularization methods with parameter *α_χ_*, despite the statistical regularization does not use any information about the SC matrix. The histograms for both parameters *α_χ_* and *α*_L_ are, however, clearly different (see Figure 3). We conclude that regularization is crucial to observe the enhancement of FC-SC similarity, and that such enhancement is, at the same time, robust with respect to the regularization parameter *α*, as far as *α* is of the same order of *α*_L_ (not orders of magnitude lower). Remarkably, these results remain qualitatively identical in a backup analysis with a supplementary extra dataset (see Supporting Information Figure S2).

### Thresholding Is Crucial for the PC FC-SC Correspondence

**Figure 4.**
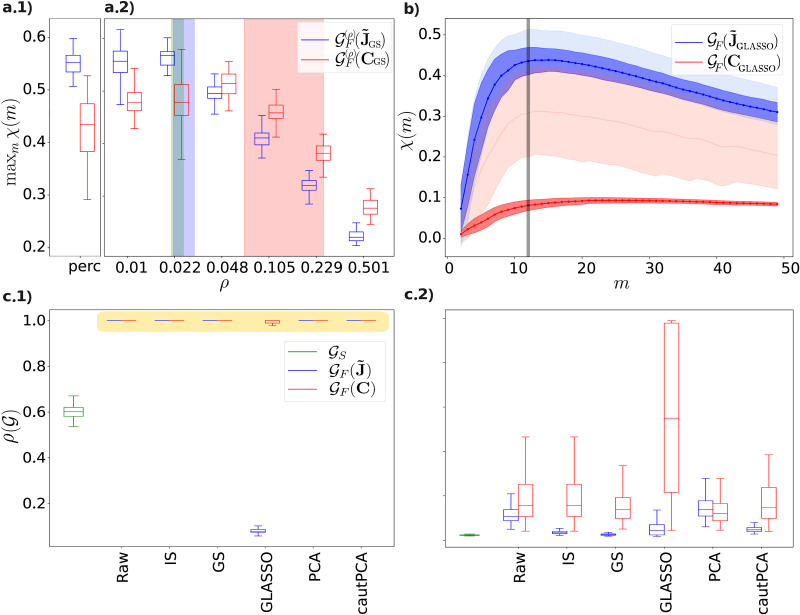
Relevance of thresholding. Subfigures A.1 and A.2 report the dependency of cross-modularity on the FC density *ρ*: the boxplots in A.2 represent the maximum (across modules) FC-SC cross-modularity, for the partial correlation–based FC $G_{F}^{\left(\rho \right)}(\tilde{J})$ (blue) and the correlation-based FC $G_{F}^{\left(\rho \right)}\left(C\right)$ (red), regularized with GS and cut at density *ρ* (while the SC graph is always cut at percolation). As a comparison, we show in A.1 (“perc”) the values that are obtained if all graphs are cut at the percolation threshold (the same as in Figure 2). We also report the average across subjects, plus/minus 1 *SD*, of the percolation density of: structural connectivity, PC-based FC, correlation-based FC (green, blue, and red vertical thick bars, respectively). These trends are robust across regularization methods. Anyway, notice that GLASSO returns a PC matrix that is much sparser than the corresponding correlation matrix (as shown in C.1). This is why in this case, if no threshold is applied, PC provides significantly higher cross-modularity curves with respect to the correlation: (B) the cross-modularity curves for **C** (red) and $\tilde{J}$ (blue), regularized with GLASSO, without any threshold (darker curves) and with percolation threshold (lighter curves) as a comparison. The boxplots C.1 and C.2 show the across-subject distribution of the single subjects’ densities $\rho \left(G\right)$ of the SC (green) and FC graphs obtained from regularized **C** (red) and $\tilde{J}$ (blue) matrices, without (C.1) and with (C.2) the cut at the percolation threshold, for different regularization strategies (*“Raw”* stands for “no regularization”). We observe how only the partial correlation regularized with GLASSO provides an FC graph that is already sparse (note that the symbols highlighted in yellow in Subfigure C.1 appearing as horizontal lines are, in fact, zero-error boxplots).

We observed that a high similarity between $G_{S}$ and $G_{F}(\tilde{J})$ only emerged provided that the latter was inferred from sparse PC matrices (see Figure 4A). It is noteworthy that thresholding disrupts the positive definiteness of the matrices, rendering the thresholded connectivity matrices noncompliant with the mathematical definition of correlation matrices. Consequently, some researchers, including Varoquaux (2019), advocate against the thresholding process. Instead, they recommend the sparsification induced by GLASSO to achieve sparsity when needed. Indeed, we have confirmed that ${\tilde{J}}_{\text{GLASSO}}$ is a sparse matrix and that further thresholding ${\tilde{J}}_{\text{GLASSO}}$ up to the percolation threshold only slightly enhances the cross-modularity of $G_{F}({\tilde{J}}_{\text{GLASSO}})$ (see Figure 4B). The same does not apply to **C**_GLASSO_, which is a dense matrix (Figure 4C).

### Relevance of FC Graphs’ Density

It is well known that a graph’s density is very relevant for many graph properties (van Wijk, Stam, & Daffertshofer, 2010). Therefore, in this section, we address the question of whether the higher FC-SC match in terms of PCs is attributable to the mere fact that the PC-based FC is sparser.

To answer this question, we estimated the FC-SC match in terms of cross-modularity, but as for fixed values of the FC network density *ρ* (tuned through thresholding), equal for correlation- and PC-based FC (while all SC graphs are cut at percolation). If $G_{F}^{\left(\rho \right)}(\tilde{J})$ exhibits a significantly higher match than $G_{F}^{\left(\rho \right)}\left(C\right)$ in a wide range of common values of *ρ*, we can conclude that the density is not the determinant factor of the phenomenon that we describe.

The results of this analysis are shown in Figure 4A.2, reporting the maximum (across modules) of the FC-SC modularity for different densities (in abscissa) of the FC graphs constructed from the regularized **C** and $\tilde{J}$ matrices. For each subject in Figure 4A.2, we regularize the **C**, $\tilde{J}$ matrices with the GS method; we then construct the associated graphs’ adjacency matrices, and threshold them up to the *x*-axis density; we finally compute the across-subjects values of *χ*, reported in the box plots. The average percolation density, plus and minus 1 *SD*, is represented in the same figure, as the three vertical bars colored in green, blue, and red for the SC, the PC-based FC and correlation-based FC, respectively. For ease of comparison, we also include in Figure 4A.1 the cross-modularity for the case in which all the matrices are cut at the so-called (subject-dependent) percolation threshold (labeled “perc”). On the one hand, we observe that sparser graphs, whose density *ρ* is more similar is to the SC density *ρ_χ_*, tend to present higher FC-SC match. On the other hand, we observe that, interestingly, the $\tilde{J}$-based match is significantly higher than the **C**-based match, for a wide range of sufficiently low values of *ρ*. This implies that we cannot simply attribute the higher PC-based FC-SC match to the fact that PC-based FC is sparser (hence, more similar in density to the SC density). See the Supporting Information for further details on the impact of graph density.

### Partition of the Population FC and Neurogenetic Interpretation

**Figure 5.**
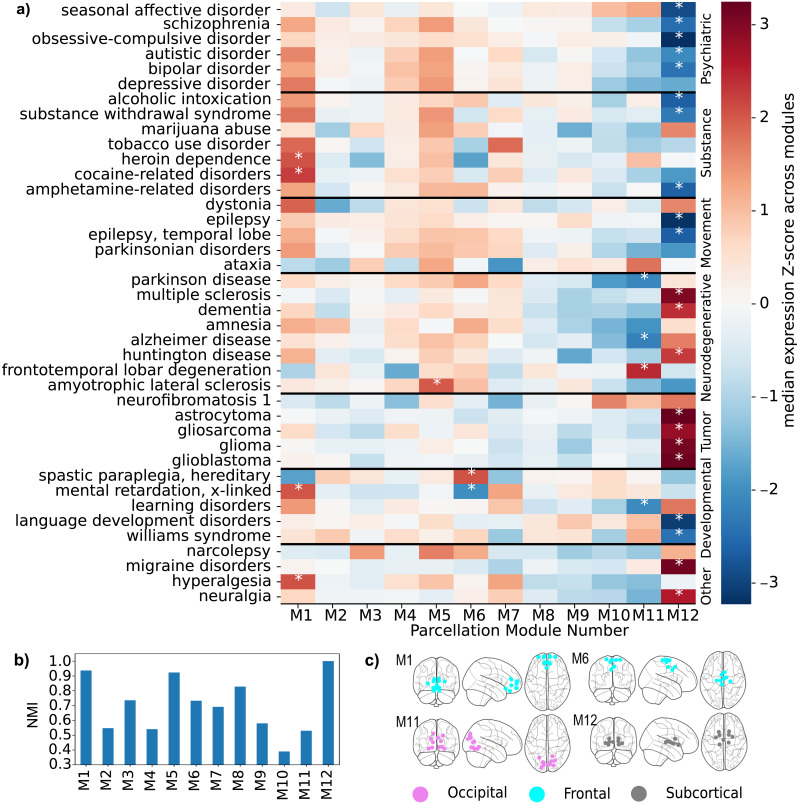
Transcriptomic expression of major brain-related disorders across different modules. For the modules of the same partition of the population $G_{F}(\tilde{J})$ illustrated in Figure 2D, we show (A) the across-modules *Z* score of the median representation of the genes associated with each disorder. Modules with absolute values higher than 2 (meaning that the disease is significantly over or underrepresented) are denoted by a white asterisk. Histogram B contains the persistence of the partition’s modules across bootstrap samplings of the dataset’s subjects; the result is expressed in terms of the average match (NMI) of each module with its most similar counterpart in each sampling’s partition. Subfigure C shows the few modules significantly associated with three or more diseases. Notice the fundamental role of module M12, which is notably present across all samplings.

Finally, we characterized the modules of the population $G_{F}(\tilde{J})$ partition shown in Figure 2 in terms of their participation in major brain-related disorders. In other words, we assessed whether some modules exhibit a significantly lower or higher expression of genes associated with a particular disease. To this end, we computed the across-modules *Z* score of the median transcriptomic expression values for sets of genes associated with each brain-related disorder across the ROIs in each module, following a procedure similar to that described in Jimenez-Marin et al. (2024). The results, shown in Figure 5, reveal that module M12, corresponding to subcortical regions, is highly relevant for most disorders. It is positively associated with tumor-related and neurodegenerative diseases and negatively associated with psychiatric, substance abuse, and movement-related ones. We additionally checked the stability of the population $G_{F}(\tilde{J})$ partition across 100 bootstrap samplings of the dataset. Indeed, we found an overall average NMI similarity score of 0.86 out of 1 between the main partition and those computed at each sampling (see the Materials and Methods section). We also found that some modules are particularly persistent: notably, module M12 appears identical in all partitions (Figure 5B).

## DISCUSSION

In the study of brain SFC, FC inferred from PC matrices shows a higher correspondence with SC, even though most previous studies have only evaluated this correspondence at the single-link level. In this work, we compared different methods to infer brain FC from resting-state BOLD time series. These methods include estimating FC from the correlation (**C**) or the PCs ($\tilde{J}$) of the time series, inferred through a regularization algorithm, and further enhancing FC graph sparsity by setting the smallest (in absolute value) matrix elements to zero, up to the so-called percolation threshold.

In general, and in line with other works (Liégeois et al., 2020; Varoquaux, 2019), we found that inferring the FC graph from regularized PC, rather than from correlation, enhances its sparsity and its similarity to the SC graph. While this is expected in the Gaussian, time-uncorrelated approximation, where the PC represents the direct interactions between brain areas (also see the Supporting Information), this result is by no means obvious in fMRI data, for several reasons. On the one hand, one expects fMRI data to be nonlinear and time-correlated and, on the other hand, the estimations of the PC-based FC are strongly influenced by the data finiteness and temporal correlations. Our article follows the above-cited studies, providing evidence of a closer FC-SC match in terms of PCs. Furthermore, we step further, by assessing: (a) the importance of a noise-cleaning or regularization method; (b) the impact of different regularization methods; and (c) the behavior of a different metrics that accounts for the similarity between FC and SC at the hierarchical-modular level (beyond the element-wise covariance between the two matrices).

The utility of having a reliable, statistically significant estimator of connectivity, similar to SC, from BOLD time series has already been pointed out, at least in the context of computational approaches to brain function. There, the brain structure is often represented in terms of latent, interpretable, inferred parameters *θ* that are eventually used as an input for machine learning classifiers or to detect differences between different groups of subjects. Such a representation is called *generative embedding* (Friston, Stephan, Montague, & Dolan, 2014; Frässle et al., 2018; Huys, Maia, & Frank, 2016; Montague, Dolan, Friston, & Dayan, 2012; Stephan & Mathys, 2014). While a standard inference method of structural parameters *θ* from the imaging data is dynamic causal modeling (DCM; Frässle et al., 2021; Rigoux & Daunizeau, 2015), a simpler alternative to DCM is the linear scheme addressed here. Albeit linear models are much simpler and less realistic than DCM, their inference could be more statistically robust for a large number of brain areas and low number of time points, as is typical of fMRI time series.

As previously noted, retrieving FC from time series data requires statistical regularization. Here, we have addressed the robustness of the FC-SC match with respect to the statistical regularization method (Figure 2), as well as with respect to the value regularization parameter, given the method. Rather interestingly, we find that the statistical regularization procedure, which has no information about SC, already brings the ensuing FC networks as similar to SC as those obtained by tuning the regularization parameter to the value *α_χ_* that maximizes the FC-SC match at the level of the single subject (Figure 3).

In this work, we observed that working with sparse graphs is crucial for observing a high degree of cross-modularity. This is why we thresholded all graphs at the so-called *percolation threshold* (also see the Materials and Methods section) that depends on the graph topology, so that no arbitrary choice regarding the final density has to be made. Some authors have argued that thresholding is not a principled approach as it results in a nonpositive definite matrix, which does not represent a valid covariance matrix and may not be invertible, thus preventing association with a Gaussian likelihood (Varoquaux, 2019). Consequently, they recommend using methods such as GLASSO to recover the adjacency matrix of $G_{F}$ as a sparse PC (a mathematically valid correlation matrix) whenever sparsity is required. Indeed, we confirmed that the GLASSO regularization, tuned by maximizing the test-set likelihood, produces a sparse PC matrix, whose cross-modularity is only slightly smaller than the one obtained by further thresholding the PC matrix, up to percolation value (Figure 4).

Given that the graphs’ density is highly relevant to the cross-modularity (as well as to many other graph properties; van Wijk et al., 2010), we analyzed this dependency (see Figure 4A) in order to rule out the hypothesis that the enhanced SC-FC match of $G_{F}(\tilde{J})$ is only due to its higher sparsity, with respect to $G_{F}\left(C\right)$. Our analysis reveals that, as a matter of fact, the higher SC-FC match for PCs occurs for a wide range of values of the density of FC connectivity matrices and is not simply induced by the relatively low density of $G_{F}(\tilde{J})$. Figure 4A also tells us that the lower the density, the higher the FC-SC match. Since the density is lowered by increasing the threshold *σ* below which we cut away the elements of the connectivity matrix, this fact suggest that higher similarity between FC and SC is obtained with the stronger links of the FC matrices.

Finally, our neurogenetics association analysis shows that Module 12, anatomically comprising the basal ganglia and thalamus—involved in motor control, cognition, and emotional regulation—has a more dominant implication across different disease groups (Figure 5). Increasing evidence suggests that gene expression patterns within the basal ganglia-thalamocortical circuitry overlap significantly with genetic profiles associated with various neuropsychiatric conditions, as well as mood and compulsive disorders. In depression, dysfunctions in the striatum and thalamus correlate with anhedonia and impaired reward processing, with genetic studies highlighting disrupted expression of serotonin transporters and dopaminergic genes (Belujon & Grace, 2017). Similarly, obsessive-compulsive disorder is associated with hyperactive cortico-striato-thalamic loops, where altered expression of glutamatergic and serotonergic genes plays a role (Grünblatt, Hauser, & Walitza, 2014). In bipolar disorder, irregularities in the basal ganglia and thalamus contribute to emotional dysregulation (Kato, 2019). In autism spectrum disorders, disruptions in synaptic excitation-inhibition imbalance and neurodevelopmental gene expression in these regions contribute to sensory-motor dysfunction and social impairment (Nelson & Valakh, 2015; Rasero et al., 2023). Our neurogenetic results also show that both the basal ganglia and thalamus are involved in seizure modulation. Indeed, the thalamus, particularly the centromedian nucleus, plays a central role in seizure propagation, with gene expression studies implicating mutations in SCN1A and GABRG2 in epileptogenesis (Lindquist, Timbie, Voskobiynyk, & Paz, 2023). On the other hand, the basal ganglia, particularly the substantia nigra pars reticulata, is involved in seizure suppression, and altered dopamine receptor gene expression has been observed in epilepsy models (Vuong & Devergnas, 2018). In Alzheimer’s disease, the thalamus and basal ganglia exhibit significant atrophy, and recent transcriptomic analyses have shown that genes such as APOE, MAPT, and PSEN1 are differentially expressed in these regions (DeTure & Dickson, 2019). Huntington’s disease, which primarily affects the striatum, is characterized by mutant HTT gene expression, leading to neuronal loss and network dysfunction between the basal ganglia and thalamus (Gatto et al., 2020).

## MATERIALS AND METHODS

### Data

Connectivity data, downloaded from the open dataset (Jimenez-Marin et al., 2023), include the preprocessed resting-state fMRI (rs-fMRI) hemodynamic BOLD time series and the DWI structural connectivity matrices of 136 healthy participants, 98 of them males, aged between 20 and 30 years old, already parceled in 183 ROIs. The rs time series include 652 time steps. Details about the preprocessing and the parcellation, from raw data of the multimodal dataset *Max Planck Institut Leipzig Mind-Brain–Body Dataset* commonly referred to as LEMON (Babayan et al., 2019), can be found in Jimenez-Marin et al. (2024).

Transcriptomic expression data, containing the expression of genes within each ROI, were also obtained from the dataset (Jimenez-Marin et al., 2024), and they are the result of the preprocessing, using the *abagen* tool (Markello et al., 2021), of the open data from the Allen Human Brain Atlas (Hawrylycz et al., 2012). In addition, we obtained the data relative to the association between genes and diseases from the archive associated with Zeighami et al. (2023).

### From Data to Brain Functional and Structural Graphs

The SC and FC graphs were recovered from the respective connectivity matrices, interpreted as adjacency matrices: each matrix entrance can be read as the strength of the connection (*link weight*) between the corresponding couple of ROIs (*nodes*). For each subject *s*, we obtained the SC directly from the structural data, while we computed multiple estimates of the FC’s connectivity matrix from the rs BOLD time series: from their regularized correlation matrix $C_{\mu }^{s}\left(\alpha_{L}\right)$ and from its inverse $J_{\mu }^{s}\left(\alpha_{L}\right)\equiv C_{\mu }^{s}{\left(\alpha_{L}\right)}^{-1}$, whose elements were next normalized as ${\tilde{J}}_{\mu ;ij}^{s}=\frac{J_{\mu ;ij}^{s}}{\sqrt{J_{\mu ;ii}^{s}J_{\mu ;jj}^{s}}}$ (PC), for each of the regularization methods *μ* that we took into account (see next section); the regularization parameter *α_L_* maximes the likelihood of the validation set, with respect to the method. The “raw,” not regularized, correlation matrix is the sample covariance of the fMRI time series; it corresponds to the sample correlation matrix when the time series are demeaned and standardized (null temporal averages and unit standard deviation) as we assume the data to be. Since there is no straightforward interpretation for negative link weights, the adjacency matrices **M** of the FC connectivity graphs were taken as the **C**, $\tilde{J}$ matrices in absolute value. More specifically, $M_{ij}=|C_{\mu }\left(\alpha_{L}\right)){}_{ij}|,|{\left({\tilde{J}}_{\mu }\left(\alpha_{L}\right)\right)}_{ij}|$, for the correlation-based and for the PC-based FC networks (i.e., $G_{F}(C_{\mu }^{s}\left(\alpha_{L}\right))$ and $G_{F}({\tilde{J}}_{\mu }^{s}\left(\alpha_{L}\right))$), respectively.

Next, we cut both structural and functional matrices **M** at the *percolation threshold* (for more detail, see the Percolation Threshold section). In the preliminary analysis, we verified that the way negative values are treated does not significantly affect the results since most of the negative elements are small in absolute value and are consequently removed in the thresholding step. At last, the thresholded matrices **M** are taken as the adjacency matrices of the SC and FC graphs of each subject *s*. At the population level, we defined the population connectivity matrix as the across-subject median of the single-subject connectivity matrices.

### Regularization Methods

Given the single-subject time series **X** (a *N* × *T* real matrix), demeaned and standardized, if *T* is finite or *q* = *N*/*T* does not vanish, the sample correlation matrix $E=\frac{{XX}^{⊺}}{T}$ and, even more, its inverse **J** = **E**^−1^ are not good estimators of the true correlation and precision matrices (Bun et al., 2017; Ibáñez-Berganza et al., 2023; Varoquaux & Craddock, 2013). Regularizing, or noise-cleaning, consists of proposing a matrix that is as similar as possible to the true correlation matrix (in particular, more than **E**).

The regularization algorithms that we took into account in this work (far from being a complete list of all the methods present in literature; Bun et al., 2017), include methods from the two most common approaches in neuroscience (i.e., the linear shrinkage approach, represented by the IS and GS, and the sparse estimator approach, such as GLASSO; Varoquaux, 2019), plus two algorithms that follow a principal component approach. All these algorithms were systematically evaluated in Ibáñez-Berganza et al. (2023) on rs-fMRI BOLD signal and synthetic data, in terms of scores such as the element-wise distance *d*(**J**, **J***^v^*) between the inferred precision **J** and the true precision **J***^v^*, and the test-set likelihood. Most of the regularization algorithms consist in regularizing the spectrum Λ of **E** while keeping the eigenvectors **U** unchanged (based on the assumption of no prior knowledge of the eigenvectors’ structure) so that the correlation matrix regularized with method *μ* has the general form **C***_μ_*(*α*) = **U**Λ(*α*)**U**^⊺^:

(a, b) LS (Ledoit & Wolf, 2004b) consists in a convex combination **C***_LS_*(*a*) = (1 − *a*)**E** + *a***T** between the sample matrix **E** and a target matrix **T** (independent of the data), such as the identity matrix $I_{N}$ (in which case we dub the method IS), or the across-subject average of the sample correlation matrix 〈**E**〉 (we call this method GS). The linear shrinkage can have different interpretations. For example, it can be seen as a trade off between bias and variance, as a shrinkage of the eigenvalues toward their grand mean (Ledoit & Wolf, 2004b), or also as an optimal Bayes estimator in the context of the Bayesian random matrix theory, choosing the minimum mean squared error as loss function and assuming a Gaussian distribution for the data and an Inverse-Wishart as prior distribution for the correlation matrix (Bun et al., 2017).

(c) PCA, also known as *eigenvalues clipping* (Bun et al., 2017), is a method that consists in considering as significant only the *p* largest eigenvalues of **E**: ${\hat{\lambda }}_{i}=\lambda_{i}$ if *i* ≤ *p*, else ${\hat{\lambda }}_{i}=\frac{\sum_{i>p}\lambda_{i}}{N-p}$.

(d) cautPCA is a simple variant of PCA that was firstly introduced in (Ibáñez-Berganza et al., 2023), where it proved to slightly outperform PCA. In this case, the spectrum is first changed as ${\hat{\lambda }}_{i}=\lambda_{p}\forall i\geq p$ and later rescaled such that *tr*[**C**] = *N*.

(e) GLASSO, in this case, **C** and **J**, are computed by maximization of the log likelihood minus a ℓ_1_ norm penalty term: **J***_GLASSO_*(*a*) = arg max*_J_* {ln*N*(**X**| **J**^−1^) − *a*∑_*i*<*j*_| *J*_*ij*_| } (Friedman et al., 2008).

(f) **ORIE**, first proposed in Ledoit and Péché (2011) and later extended in Bun, Allez, Bouchaud, and Potters (2016), is a method that consists of correcting the eigenvalues of the sample estimator **E** as $\lambda_{k}^{\prime }=\frac{\lambda_{k}}{{\left|1-q+{qz}_{k}s\left(z_{k}\right)\right|}^{2}}$ where $s\left(z\right)=Tr\left[{\left(zI_{N}-E\right)}^{-1}\right]/N$, and *z*_*k*_ = *λ_k_* − *iη* (*i* is the imaginary unit; Bun, Allez, et al., 2016; Bun, Bouchaud, & Potters, 2016; Bun et al., 2017; Ibáñez-Berganza et al., 2023; Ledoit & Péché, 2011). This estimator minimizes, among all estimators that share the same eigenvectors of the sample correlation, the Euclidean distance from the true correlation matrix in the high-dimensional limit. The parameter *η* should be small, and such that *Nη* ≫ 1 (Bun, Bouchaud, & Potters, 2016). While a convenient choice is given by *η* = *N*^−1/2^ (Bun, Allez, et al., 2016; Bun, Bouchaud, & Potters, 2016; Ibáñez-Berganza et al., 2023), this may lead to values that are not small enough if *N* is not extremely large (Bun, Bouchaud, & Potters, 2016). Therefore, following Ibáñez-Berganza et al. (2023), we choose to cross-validate this parameter to maximize the validation-set likelihood.

Except for ORIE and GLASSO, the regularization methods that we took into account in this work depend on a tuning parameter that we generically call *α*, ranging from zero (corresponding to no regularization at all, in which case the correlation matrix estimate equates the sample correlation **E**) to *α* = 1 (maximum regularization). The regularization parameter coincides with the parameter *a* of LS and with $\frac{N-p}{N}$ for PCA and cautPCA.

As explained in more detail in the Supporting Information, the regularization parameters are set, by cross-validation, to those maximizing the validation-set likelihood at the level of the single subject BOLD time series.

### Percolation Threshold

As mentioned, we obtained the connectivity graphs from the respective thresholded connectivity matrices. In particular, we thresholded the matrices at the percolation cutoff, meaning that we gradually removed the matrix elements in order of increasing weight, stopping just before the corresponding graph would break into more than one disconnected component. Among the many sparsification methods that have been proposed in neuroscience, this method has the advantage of providing a connected graph whose final density depends on the graph topology. Moreover, in Nicolini et al. (2020), the application of the percolation threshold was shown to provide the optimal balance between the removal of noise and genuine information, maximizing the distance of FC from its randomized counterpart, and it was suggested that its application is critical for the extraction of the large-scale structure from the network.

### Hierarchical Clustering

We compared the architectures of each pair of SC and FC graphs, both at the subject and population levels, based on their hierarchical clusterings. Hierarchical, or agglomerative, clustering is an unsupervised method for finding communities in *N*-dimensional observation vectors. The algorithm produces a whole hierarchy of nested data partitions that can be represented as a dendrogram. As a result of this step, we obtained a couple of sets of partitions $P_{G_{F}}\left(m\right)$ and $P_{G_{S}}\left(m\right)$ in *m* modules, ∀*m* = 2, ⋯50, representing the hierarchical partitions of both FC and SC graphs.

In particular, we have made use of a hierarchical clustering algorithm whose distance matrix is built as exp(−**M***_ij_*), where **M** is the connectivity graph adjacency matrix. More in detail, we used SciPy implementation module (Virtanen et al., 2020), with metric = cosine metric and method = weighted, taking as input the so called *1-D condensed distance matrix* built from the adjacency matrix.

### Cross-Modularity

To evaluate the similarity of the partitions of the FC and SC, we introduced a metric that, given the partitions $P_{G_{F}}\left(m\right)$ and $P_{G_{S}}\left(m\right)$, simultaneously takes into account their individual quality and their reciprocal similarity for each number of modules *m*. Therefore, we define the cross-modularity *χ*(*m*) as

$$\chi_{G_{F},G_{S}}\left(m\right)={\left(Q_{G_{F}}\left(m\right)\cdot Q_{G_{S}}\left(m\right)\cdot \nu_{G_{F},G_{S}}\left(m\right)\right)}^{1/3}$$ (2)

where $Q_{G}\left(m\right)$ is the quality of partition $P_{G}\left(m\right)$, measured in terms of the Newmann Modularity (Newman, 2006; we measured it ignoring the links’ weights), and $\nu_{G_{F},G_{S}}\left(m\right)$ is the similarity of partitions $P_{G_{F}}\left(m\right)$ and $P_{G_{S}}\left(m\right)$, measured in terms of adjusted NMI (Xuan Vinh et al., 2010). Cross-modularity is a variation of the homonym metric first introduced in Diez et al. (2015); there, a unique partition *P*(*m*) was applied to both SC and FC graphs, and the agreement between the communities of the two graphs was then computed as the average agreement (measured as Sorensen index) between the couple of communities induced on SC and FC by each module of *P*(*m*).

More in detail, Newman modularity is a measure of the quality of a particular partition of a graph into modules, defined in Newman (2006) as:

$$Q_{G}=\frac{1}{2L}\sum_{i,j}^{N}\left(A_{ij}-\frac{d_{i}d_{j}}{2L}\right)\delta \left(c_{i},c_{j}\right)$$ (3)

where *L* is the number of edges, *A* is the adjacency matrix, *d*_*i*_ is the degree of *i*, and *δ*(*c*_*i*_, *c*_*j*_) is 1 if *i* and *j* belong to the same community, else 0. This quantity is proportional to the number of intramodule edges minus its expected number in a network with equal degree sequence and random links. Consequently, modularity approaching 1 indicates a high-quality partition (high intracluster and low intercluster density), while it would vanish in the case of a random one (Van Mieghem et al., 2010).

Mutual information (MI) is an information-theoretic tool that measures the amount of information shared by two partitions; its normalized and adjusted version (NMI), where the adjustment discards the matches due to chance, was proposed in Romano et al., 2016):

$$\;NMI=\frac{MI-E\left[MI\right]}{\max\;MI-E\left[MI\right]}$$ (4)

(where symbol *E*[⋅] stands for the expected value), so that this quantity vanishes in case of comparison of random partitions and reaches 1 for perfect coherence.

As a consequence, cross-modularity may achieve a maximum value of 1 in the case of high-quality partitions and a perfect match, while it vanishes in the case of unrelated partitions or if at least one of them is random. We have measured Newman modularity using the implementation in Hagberg et al. (2008) and NMI using the implementation in Pedregosa et al. (2011).

### Spectral Distance

In addition to the single-subject cross-modularity, we also computed the spectral distance (Jovanović & Stanić, 2012) of the connectivity graphs’ adjacency matrices between each couple of subjects for both the SC graph and every estimate of the FC graph. In general, the spectral distance measures the difference between a couple of matrices in terms of the difference between their eigenvalues:

$$d\left(M^{\left(1\right)},M^{\left(2\right)}\right)=\sum_{i}^{N}|\lambda_{i}^{\left(1\right)}-\lambda_{i}^{\left(2\right)}|$$ (5)

where $\lambda_{i}^{\left(j\right)}$, with *j* = 1, 2 and *i* = 1, …, *N* are the matrices **M**^(1)^ and **M**^(2)^ eigenvalues. To make fair the comparison between the spectral distance of the subjects’ FC (which are bounded, in absolute value, between 0 and 1) and SC (which are expressed as the number of white matter streamlines) adjacency matrices, we normalized the values of the (thresholded) $G_{S}$ adjacency matrix as $\frac{2\arctan\left(\cdot \right)}{\pi }$.

### Characterization of the Optimal FC Brain Partition in Terms of Brain-Related Disorders

Once obtained the optimal (in the sense of higher cross-modularity) partition of the optimal (in the sense of statistical regularization) estimate of the FC graph at the population level, we characterized its modules depending on their participation in 40 major brain-related disorders from seven disease groups (psychiatric disorders, substance abuse, movement disorders, neurodegenerative diseases, tumor conditions, developmental disorders, and others). Our method follows the procedure adopted in Jimenez-Marin et al. (2024), work associated with the Zenodo dataset from which we extracted the transcriptomic data. For each disease and for each module, we computed the disease expression value as the median value across the genes associated with the disease and across the nodes contained in the module. The heatmap in Figure 5 shows the across-modules *Z* scores of the diseases expressions, with absolute values higher than 2 marked with an asterisk, and diseased grouped by the World Health Organization categories (also obtained from archive; Zeighami et al., 2023).

### Robustness Check of the Optimal FC Brain Partition

We have checked the robustness of the partition of our estimate of the population FC graph by comparing it with different estimates inferred from 100 bootstrap samplings of the dataset. In particular, for 100 times, we iterated the following procedure: We randomly selected 136 subjects (importantly, with repetitions), we computed an estimate of the population $G_{F}(\tilde{J})$ of these subjects, with regularization method GS and we partitioned it into 12 modules; finally, we computed the match of each partition with the whole-dataset one (i.e., the partition shown in Figure 2) in terms of NMI, finding an overall score of 0.86. We additionally measured the robustness of each whole-dataset partition’s module as its across-sampling average NMI similarity to the most similar module in the subsampling partition. The results, shown in Figure 5B, reveal that some modules, Module M12 in primis, are particularly robust.

## ACKNOWLEDGMENTS

“This work has been supported by: the European Union under the scheme HORIZON-INFRA-2021-DEV-02-01 – Preparatory phase of new ESFRI research infrastructure projects, Grant Agreement n.101079043, “SoBigData RI PPP: SoBigData RI Preparatory Phase Project”; by the project “Reconstruction, Resilience and Recovery of Socio-Economic Networks” RECON-NET EP\_FAIR\_005 - PE0000013 “FAIR” - PNRR M4C2 Investment 1.3, financed by the European Union – NextGenerationEU; by the European Union - Horizon 2020 Program under the scheme ‘INFRAIA-01-2018-2019 - Integrating Activities for Advanced Communities’, Grant Agreement n.871042, ‘SoBigData++: European Integrated Infrastructure for Social Mining and Big Data Analytics’ (www.sobigdata.eu); Grant No. PID2023-149174NB-I00 financed by MICIU/AEI/10.13039/501100011033 and EDRF/EU funds”.

Jesus M. Cortes acknowledges financial support from Ikerbasque: The Basque Foundation for Science, and from Spanish Ministry of Science (PID2023-148012OB-I00), Spanish Ministry of Health (PI22/01118), Basque Ministry of Health (2023111002 & 2022111031).

## SUPPORTING INFORMATION

Supporting information for this article is available at https://doi.org/10.1162/NETN.a.22.

## AUTHOR CONTRIBUTIONS

Francesca Santucci: Conceptualization; Formal analysis; Investigation; Methodology; Software; Validation; Visualization; Writing – original draft; Writing – review & editing. Antonio Jimenez-Marin: Data curation; Resources; Software. Andrea Gabrielli: Conceptualization; Supervision; Writing – review & editing. Paolo Bonifazi: Conceptualization; Writing – review & editing. Miguel Ibáñez-Berganza: Conceptualization; Methodology; Supervision; Writing – review & editing. Tommaso Gili: Conceptualization; Supervision; Writing – review & editing. Jesus M. Cortes: Conceptualization; Data curation; Methodology; Supervision; Visualization; Writing – original draft; Writing – review & editing.

## FUNDING INFORMATION

Jesus M. Cortes, Ikerbasque, Basque Foundation for Science (https://dx.doi.org/10.13039/501100003989), Award ID: 202211103 and 2023111002. Miguel Ibáñez-Berganza, Spanish Ministry and Agencia Estatal de Investigación (AEI), Award ID: Project I + D + i Ref. No. PID2020-113681GB-I00. Tommaso Gili, European Union - NextGenerationEU - National Recovery and Resilience Plan (Piano Nazionale di Ripresa e Resilienza, PNRR), project “SoBigData.it - Strengthening the Italian RI for Social Mining and Big Data Analytics,” Award ID: Grant IR0000013 (No. 3264, 28/12/2021). Francesca Santucci, European Union - NextGenerationEU - National Recovery and Resilience Plan (Piano Nazionale di Ripresa e Resilienza, PNRR), project “SoBigData.it - Strengthening the Italian RI for Social Mining and Big Data Analytics,” Award ID: Grant IR0000013 (No. 3264, 28/12/2021). Miguel Ibáñez-Berganza, European Union - NextGenerationEU - National Recovery and Resilience Plan (Piano Nazionale di Ripresa e Resilienza, PNRR), project “SoBigData.it - Strengthening the Italian RI for Social Mining and Big Data Analytics,” Award ID: Grant IR0000013 (No. 3264, 28/12/2021). Andrea Gabrielli, Museo Storico della Fisica e Centro Studi e Ricerche Enrico Fermi (https://dx.doi.org/10.13039/501100014144). Tommaso Gili, Scuola IMT Alti Studi Lucca (https://dx.doi.org/10.13039/100015963).

## Supplementary Material



## TECHNICAL TERMS

Functional connectivity (FC): The generic network of activity patterns between brain areas.
Structural connectivity (SC): The generic network of anatomical connections between brain areas.
SC graph $G_{S}$: The graph representing SC, here built from thresholded DWI data.
FC graph $G_{F}$: The graph representing FC, here inferred both as (regularized and thresholded) correlation and partial correlation matrices, from rs-fMRI data.
Precision matrix **J**: The matrix inverse of the (possibly regularized) correlation matrix: **J** = **C**^−1^.
Sample correlation matrix **E**: The unbiased (“raw”) estimator of the true correlation matrix, computed from the standardized *N* × *T* data matrix **X** as **E** = **XX**^T^/*T*.
Regularized correlation matrix **C**_*μ*_(*α*): A denoised estimate of the true correlation matrix, using method *μ* and tuning parameter *α*.
Regularized partial correlation (PC) matrix ${\tilde{J}}_{\mu }$(*α*): The normalized version of the precision matrix. In a Gaussian approximation, it accounts for direct dependencies only.
