# Supplementary material for: Partial correlation as a tool for mapping functional-structural correspondence in human brain connectivity
Source: Netw Neurosci. 2025 Sep 19;9(3):1065–86. doi: 10.1162/NETN.a.22 (PMC12548666; doi:10.1162/NETN.a.22)
Supplement: Supplementary file 1 [file netn-9-3-1065-s001.pdf]

Supplementary Information for "Partial Correlation as a Tool for Mapping Functional-Structural Correspondence in Human Brain Connectivity"

Francesca Santucci[1,2,*], Antonio Jimenez-Marin[2], Andrea Gabrielli[3,4,5], Paolo Bonifazi[2,6,7], Miguel Ibáñez-Berganza[1,8], Tommaso Gili[1], and Jesus M. Cortes[2,6,9]

[1]NETWORKS Unit, IMT School for Advanced Studies, Lucca, Italy
[2]Computational Neuroimaging Lab, Biocruces-Bizkaia HRI, Barakaldo, Spain
[3]Dipartimento di Ingegneria Civile, Informatica e delle Tecnologie Aeronautiche, Università degli Studi Roma Tre, Rome, Italy
[4]"Enrico Fermi" Research Center - CREF, Rome, Italy
[5]Istituto dei Sistemi Complessi - CNR, Rome, Italy
[6]Ikerbasque: The Basque Foundation for Science, Bilbao, Spain
[7]Department of Physics and Astronomy, University of Bologna, Bologna, 40127, Italy.
[8]INdAM-GNAMPA Istituto Nazionale di Alta Matematica "Francesco Severi", P.le Aldo Moro 5, 00185 Rome, Italy
[9]Department of Cell Biology and Histology. University of the Basque Country, Spain
[*]Correspondence to: francesca.santucci@imtlucca.it

1 Relevance of FC graphs' density

For each subject in Fig. 4-**a.2** in the main text, we regularize the **C**, $\tilde{\mathbf{J}}$ with the GS strategy; we then construct the associated graphs' adjacency matrices, and threshold them up to the x-axis density; we finally compute the across-subjects values of χ, reported in the box-plots. The average percolation density, plus and minus one standard deviation, is represented in the same figure, as the three vertical bars colored in green, blue and red for the SC, the partial correlation-based FC and the correlation-based FC, respectively. For ease of comparison, we also include in Fig. 4-**a.1** the cross-modularity for the case in which all the matrices are cut at the so-called (subject-dependent) percolation threshold (labeled "perc"). The results of Fig. 4-**a.2** are qualitatively identical when we threshold the SC at the same density as the FC and when we use different regularization criteria.

2 Statistical Regularization

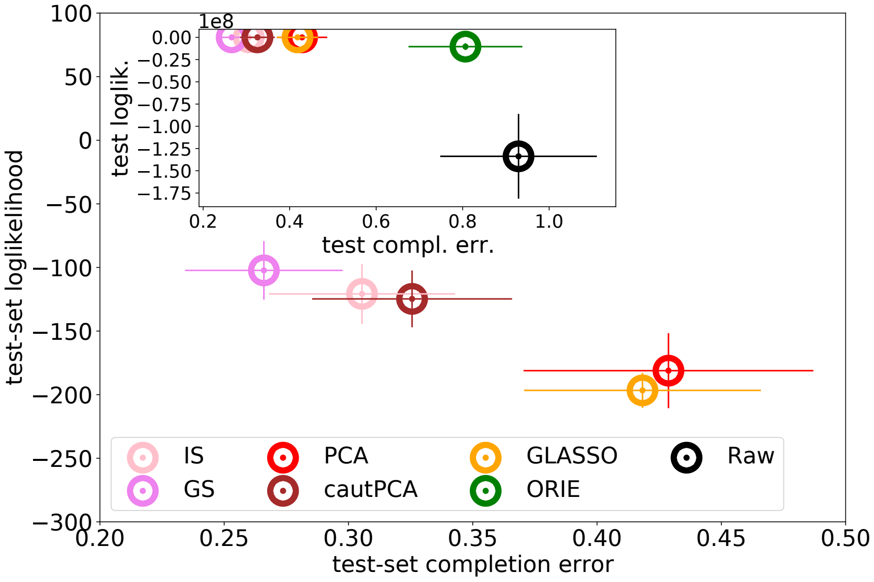

Figure S1: **Values of the test-set likelihood.** Scores of the regularized correlation (and hence precision) matrices, computed on the training set, in terms of test-set likelihood and completion error (see Ibáñez-Berganza et al. (2023)), for different regularization methods. "Raw" stands for no regularization. Importantly, for each subject, the training and test sets are obtained randomly splitting the time series (time-wise), without altering the temporal order of the vectors.

The regularization methods that we took into account rely on a tuning parameter α. Its optimal value α_L is set by maximization of the log-likelihood $\ln(P(\mathbf{X}_v|\mathbf{C}_{tr}))$ evaluated on the *validation-set* $\mathbf{X}_v$, different from the *pure-training-set* $\mathbf{X}_{tr}$ used to infer $\mathbf{C}_{tr}$ (we adopt the notation of reference (Ibáñez-Berganza et al. (2023)), in which $\mathbf{X} = ((\mathbf{X}_{tr}, \mathbf{X}_v), \mathbf{X}_{te})$, where the joint pure-training and validation-sets $(\mathbf{X}_{tr}, \mathbf{X}_v)$ constitute the training-set, and where "te" refers to test-set). We perform the maximization of the validation-set likelihood across a grid of values of α through k-fold cross-validation (Pedregosa et al. (2011)). The training set $(\mathbf{X}_{tr}, \mathbf{X}_v)$ is randomly split into k smaller sets, or folds; at each of the k iterations, $k-1$ folds are taken as pure-training set $(\mathbf{X}_{tr})$, and the

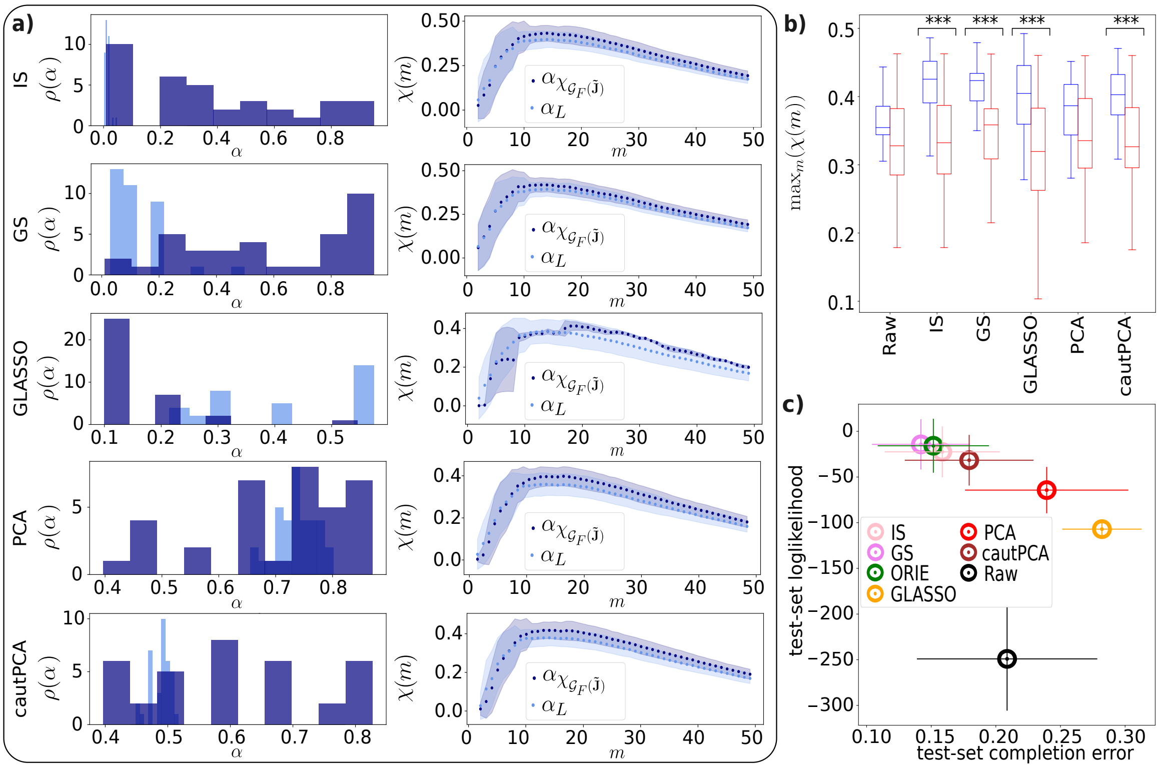

Figure S2: **Regularization tuning parameters** (subfigure **a**) and maximum cross-modularity (subfigure **b**) computed on a **supplementary extra dataset**, and **test-set likelihood scores** (subfigure **c**). The extra dataset is composed of 35 subjects, with 116 ROIs and rs-BOLD time series of 180 time steps. Coherently with our main results (see Fig. 3), we do not observe any significant gap between the cross-modularity of $\mathcal{G}_F(\tilde{\mathbf{J}}_\mu(\alpha))$ choosing the tuning parameter α_L that maximizes the validation-set likelihood and $\alpha_{\chi_{\mathcal{G}_F(\tilde{\mathbf{J}}_\mu)}}$ that maximizes the cross-modularity of $\mathcal{G}_F(\tilde{\mathbf{J}}_\mu)$ (subfig. **a**). In addition, the subjects' $\chi(m)$ curves exhibit a maximum at approximately the same value of m as in the main dataset (subfig. **a**). As in the case of the main dataset, the cross-modularity of the FC graph retrieved from regularized partial correlation is significantly higher than that retrieved from correlation (subfig. **b**; also compare with Fig. 2 in the main text. The three asterisks indicate the methods corresponding to a p-value lower than 10^{-3} for the Mann-Whitney U rank test of the null hypothesis that the distribution underlying the sample corresponding to $\mathbf{C}$ is stochastically less than the distribution underlying the sample corresponding to $\tilde{\mathbf{J}}$).

remaining one as validation-set ($\mathbf{X}_v$); finally, each parameter's score is computed as the average of the iteration scores. In Fig. S1 we show the test-set likelihood of the main dataset corresponding to each regularization method. The statistical regularization methods were implemented using the repository (Lucibello (2023)).

An important detail regarding the cross-validation procedure is that we respect the original temporal order of the series (the index t of X_{jt}) in each fold of the pure-training-set/validation-set division. We use, in other terms, the flag `shuffle=False` of the function `split_train_test` of (Lucibello (2023)). In the presence of a high correlation time (as the one exhibited by the dataset

analysed in the main text), we notice that it is necessary not to shuffle the temporal order of the data in the maximization of the validation-set likelihood by cross-validation. In short, if the correlation time of the data is of the same order of the number of samples in the validation-set, and if one shuffles the temporal order, the validation-set likelihood can be strongly overestimated for low values of the regularization parameter, since the validation-set vectors are too similar to some vectors in the pure-training-set. As a consequence, the consequent values of α_L may be severely underestimated. As a consistency check of these arguments, we have verified that in the supplementary extra dataset (see Fig. S2), which exhibits a significantly lower correlation time, shuffling or not shuffling the temporal order leads to qualitatively identical results (in their turn, consistent with the results found for our main dataset, without shuffling), also in terms of FC-SC cross-modularity. This point will be explained in detail in an upcoming publication.

3 Consistency analysis for the supplementary dataset

We performed a consistency analysis of the main article results in a supplementary dataset. The supplementary dataset consists the rs-fMRI BOLD series and DWI matrices of 35 subjects, with $N = 116$ ROIs, and $T = 180$ time steps (please, see the details in (Mastrandrea et al. (2017)). Fig. S2 summarizes our results (compare with Figs. 2, 3). We observe that the article results are perfectly consistent across datasets.

4 FC-SC correspondence at the population level

We show here in detail the effect of the regularization on $\chi(m)$ at the population level, having selected the simple GS as reference method. Figs. S3-**a-c** provide a representation of the population $\mathcal{G}_F(\mathbf{C})$, $\mathcal{G}_F(\tilde{\mathbf{J}})$ and $\mathcal{G}_S$. From a visual inspection, $\mathcal{G}_F(\tilde{\mathbf{J}})$ appears characterized by a higher modularity than $\mathcal{G}_F(\mathbf{C})$ and by a lower density, closer to that of $\mathcal{G}_S$.

Figs. S3-**d,e** reveal that $\tilde{\mathbf{J}}$ provides a higher cross-modularity than $\mathbf{C}$, but the effect of regularization is less significant compared to the single-subject case.

5 Relation between Structure and Function in the linear approximation

In this section, we provide a detailed discussion about why we expect partial correlations to be closer (than simple correlations) to structure, and what are the working hypothesis underpinning this expectation.

The working hypothesis of this and other articles considering full and partial covariance matrices is a linear interaction between different brain areas (see, for example, Liégeois, Santos, Matta, Van De Ville, and Sayed (2020); Ryali, Chen, Supekar, and Menon (2012); Varoquaux, Gramfort, Poline, and Thirion (2010)). In other words, the hypothesis is that the time series' stationary probability distribution is normally distributed with a certain correlation matrix $\mathbf{C}$ (and associated precision matrix $\mathbf{J} = \mathbf{C}^{-1}$), to be inferred:

$$P(\mathbf{X};\mathbf{J}) = \frac{|\mathbf{J}|^{T/2}}{(2\pi)^{NT/2}} \exp\{-\frac{T}{2}\mathrm{tr}[\mathbf{E}\cdot\mathbf{J}]\} \quad (1)$$

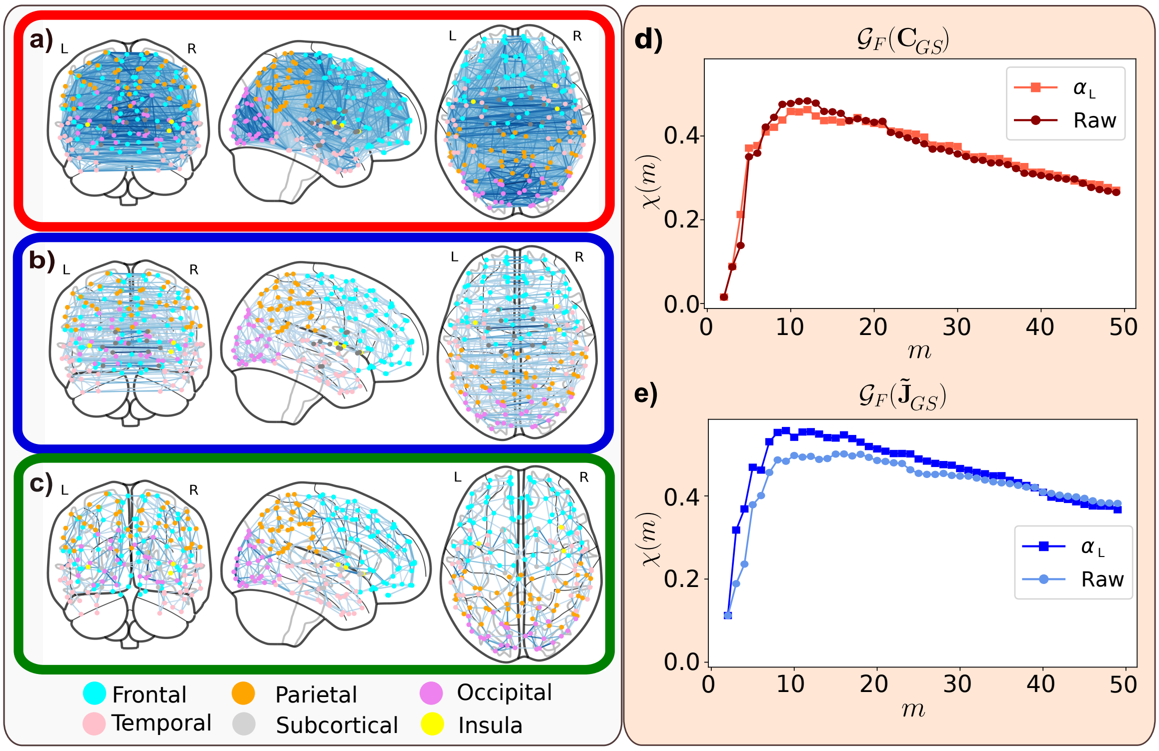

Figure S3: **Connectivity graphs, inferred from C and $\tilde{\mathbf{J}}$, at the population level**. The first column reveals the aspect of the population brain graphs, where darker edges correspond to stronger links: $\mathcal{G}_F(\mathbf{C})$ (**a**, red frame), $\mathcal{G}_F(\tilde{\mathbf{J}})$ (**b**, blue frame) (both computed as the median of the single subject matrices regularized with GS), and $\mathcal{G}_S$ (**c**, green frame). A visual inspection appears in agreement with the rest of the results, suggesting that $\mathcal{G}_F(\tilde{\mathbf{J}})$ is closer to $\mathcal{G}_S$ than $\mathcal{G}_F(\mathbf{C})$. The second column shows the population cross-modularity $\chi(m)$, for $\mathcal{G}_F(\mathbf{C})$ (**d**, red) and $\mathcal{G}_F(\tilde{\mathbf{J}})$ (**e**, blue), for not regularized ("Raw") and regularized (α_L) matrices.

where (we assume the time series $\mathbf{X}$ to be demeaned and standardized)

$$E_{ij} := \frac{1}{T}\sum_{t=1}^{\mathsf{T}} X_{it}X_{jt} = \frac{1}{T}(\mathbf{X}\mathbf{X}^{\mathsf{T}})_{ij} \tag{2}$$

is the sample covariance, or, equivalently,

$$P(\mathbf{X}|\mathbf{J}) = \prod_{t=1}^{T} \mathcal{N}(\boldsymbol{x}_t;\mathbf{J}) \tag{3}$$

where

$$\mathcal{N}(\boldsymbol{x};\mathbf{J}) := \frac{|\mathbf{J}|^{1/2}}{(2\pi)^{N/2}} \exp\{-\frac{1}{2}\boldsymbol{x}^{\mathsf{T}}\mathbf{J}\boldsymbol{x}]\} \tag{4}$$

is the multivariate normal distribution and where

$$C_{ij} := \int [d\boldsymbol{x}] x_i x_j \,\mathcal{N}(\boldsymbol{x};\mathbf{J}) = (\mathbf{J}^{-1})_{ij} \tag{5}$$

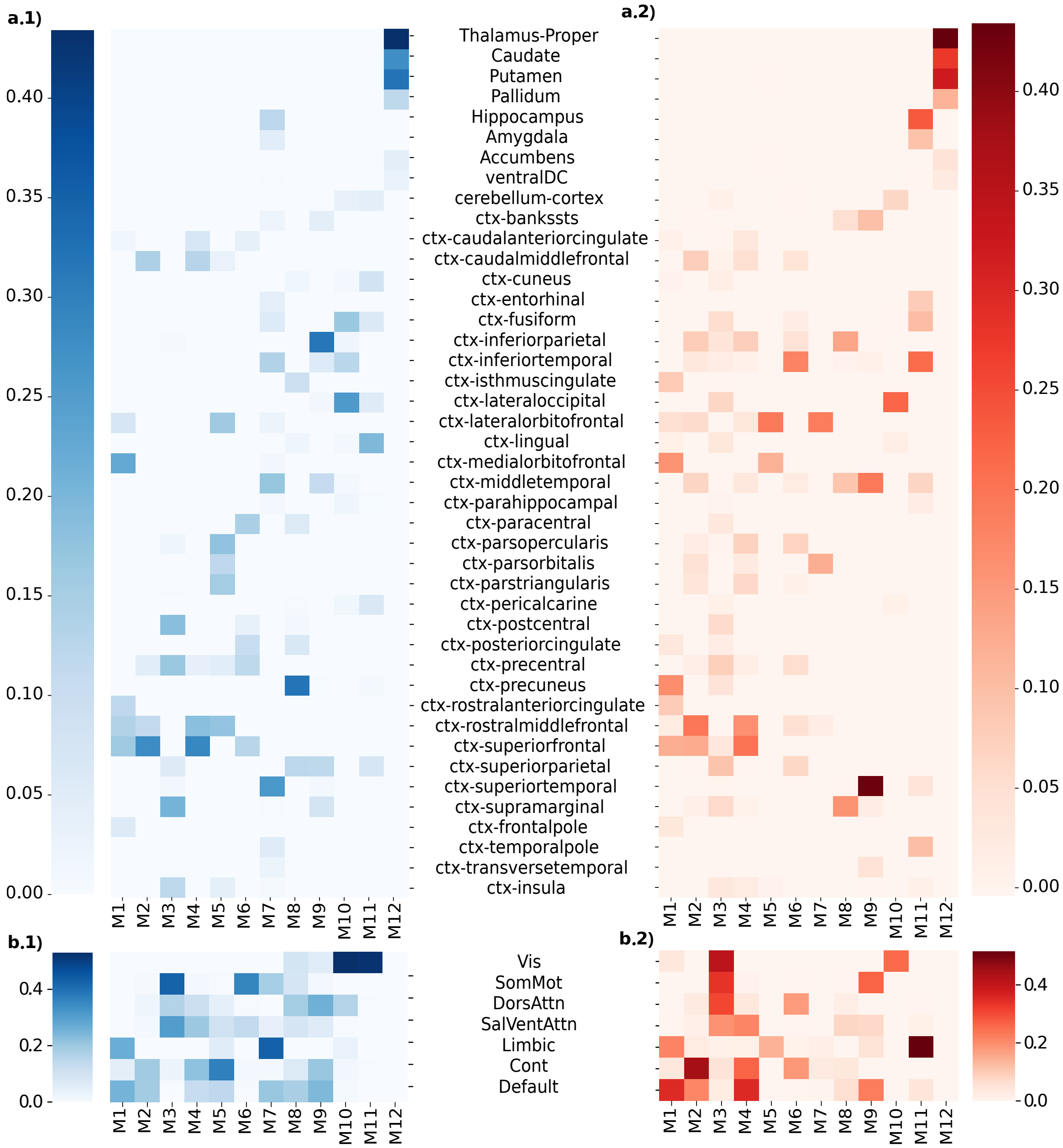

Figure S4: **Overlap between modules and brain areas**. This image shows the overlap between each module of both the population $\mathcal{G}_F(\mathbf{C})$ (red) and $\mathcal{G}_F(\tilde{\mathbf{J}})$ (blue), regularized with GS (in the case of $\mathcal{G}_F(\tilde{\mathbf{J}})$ the modules are the same as those shown in Fig. 2 in the main text), the Desikan-Killany structural areas (subfigures a), and the Yeo functional areas (subfigures b). The overlap is measured in terms of the Jaccard index between each couple module-area's volumes.

This assumption provides a probabilistic relation $P(\mathbf{X}|\mathbf{J})$ between different areas' time series $\mathbf{X}$, induced by the precision matrix $\mathbf{J}$ that, in a statistical physics language, represents the (harmonic) interactions between couples of brain areas. More in detail, x_i and x_j represent the deviations of areas i and j from their respective baseline mean BOLD activities, coupled by causal, harmonic interactions (i.e., by a virtual spring with elastic constant J_{ij}, to be inferred) that induce an elastic interaction energy equal to $\epsilon_{ij} = x_i x_j J_{ij}$. Thus, $P(\mathbf{X}|\mathbf{J})$ corresponds to the Bolztmann probability distribution associated with the energy induced by the "structural" constraints $\mathbf{J}$. Such an interaction matrix induces, in turn, the covariances $\mathbf{C}$. Therefore, $\mathbf{J}$ represents the direct, causal interactions, while $\mathbf{C}$ represents the emerging (direct and spurious) covariances, associated with a probability distribution $P(\mathbf{X}|\mathbf{J})$ over the ensemble of all possible configurations of BOLD activity. Therefore, in a linear approximation, the relation between structural connections $\mathbf{J}$ and associated, emerging covariances $\mathbf{C}$ is simply $\mathbf{C} = \mathbf{J}^{-1}$.

References

Ibáñez-Berganza, M., Lucibello, C., Santucci, F., Gili, T., & Gabrielli, A. (2023, August). Noise cleaning the precision matrix of short time series. *Physical Review E*, *108*(2). (Publisher: American Physical Society) doi: 10.1103/PhysRevE.108.024313

Liégeois, R., Santos, A., Matta, V., Van De Ville, D., & Sayed, A. H. (2020, November). Revisiting correlation-based functional connectivity and its relationship with structural connectivity. *Network Neuroscience*, *4*(4), 1235–1251. Retrieved from https://direct.mit.edu/netn/article/4/4/1235/95855/Revisiting-correlation-based-functional (Publisher: MIT Press) doi: 10.1162/NETN_A_00166

Lucibello, C. (2023, October). *CarloLucibello/covariance-estimators.* Retrieved 2025-03-04, from https://github.com/CarloLucibello/covariance-estimators (original-date: 2021-09-30T14:49:37Z)

Mastrandrea, R., Gabrielli, A., Piras, F., Spalletta, G., Caldarelli, G., & Gili, T. (2017, July). Organization and hierarchy of the human functional brain network lead to a chain-like core. *Scientific Reports*, *7*(1), 4888. Retrieved 2025-03-04, from https://www.nature.com/articles/s41598-017-04716-3 (Publisher: Nature Publishing Group) doi: 10.1038/s41598-017-04716-3

Pedregosa, F., Varoquaux, G., Gramfort, A., Michel, V., Thirion, B., Grisel, O., ... Duchesnay, \. (2011). Scikit-learn: Machine Learning in Python. *Journal of Machine Learning Research*, *12*, 2825–2830. Retrieved from http://scikit-learn.sourceforge.net.

Ryali, S., Chen, T., Supekar, K., & Menon, V. (2012, February). Estimation of functional connectivity in fMRI data using stability selection-based sparse partial correlation with elastic net penalty. *NeuroImage*, *59*(4), 3852–3861. doi: 10.1016/j.neuroimage.2011.11.054

Varoquaux, G., Gramfort, A., Poline, J. B., & Thirion, B. (2010, August). Brain covariance selection: better individual functional connectivity models using population prior. *Advances in neural information processing systems*, *23*. Retrieved from http://arxiv.org/abs/1008.5071
